# 3-D DNA methylation phenotypes correlate with cytotoxicity levels in prostate and liver cancer cell models

**DOI:** 10.1186/2050-6511-14-11

**Published:** 2013-02-11

**Authors:** Arkadiusz Gertych, Jin Ho Oh, Kolja A Wawrowsky, Daniel J Weisenberger, Jian Tajbakhsh

**Affiliations:** 1 Translational Cytomics Group, Department of Surgery, Cedars-Sinai Medical Center, Los Angeles, CA, 90048, USA; 2 Chromatin Biology Laboratory, Department of Surgery, Cedars-Sinai Medical Center, Los Angeles, CA, 90048, USA; 3 Bioinformatics Laboratory, Department of Surgery, Cedars-Sinai Medical Center, Los Angeles, CA, 90048, USA; 4 Department of Biomedical Sciences, Cedars-Sinai Medical Center, Los Angeles, CA, 90048, USA; 5 USC Epigenome Center, Keck School of Medicine, University of Southern California, Los Angeles, CA, 90089, USA

**Keywords:** DNA methylation phenotype, Chromatin distribution, High-throughput cell assay, 3D image analysis, MethyLight, Repetitive element, Epigenetic drug

## Abstract

**Background:**

The spatial organization of the genome is being evaluated as a novel indicator of toxicity in conjunction with drug-induced global DNA hypomethylation and concurrent chromatin reorganization. 3D quantitative DNA methylation imaging (3D-qDMI) was applied as a cell-by-cell high-throughput approach to investigate this matter by assessing genome topology through represented immunofluorescent nuclear distribution patterns of 5-methylcytosine (MeC) and global DNA (4,6-diamidino-2-phenylindole = DAPI) in labeled nuclei.

**Methods:**

Differential progression of global DNA hypomethylation was studied by comparatively dosing zebularine (ZEB) and 5-azacytidine (AZA). Treated and untreated (control) human prostate and liver cancer cells were subjected to confocal scanning microscopy and dedicated 3D image analysis for the following features: differential nuclear MeC/DAPI load and codistribution patterns, cell similarity based on these patterns, and corresponding differences in the topology of low-intensity MeC (LIM) and low in intensity DAPI (LID) sites.

**Results:**

Both agents generated a high fraction of similar MeC phenotypes across applied concentrations. ZEB exerted similar effects at 10–100-fold higher drug concentrations than its AZA analogue: concentration-dependent progression of global cytosine demethylation, validated by measuring differential MeC levels in repeat sequences using MethyLight, and the concurrent increase in nuclear LIM densities correlated with cellular growth reduction and cytotoxicity.

**Conclusions:**

3D-qDMI demonstrated the capability of quantitating dose-dependent drug-induced spatial progression of DNA demethylation in cell nuclei, independent from interphase cell-cycle stages and in conjunction with cytotoxicity. The results support the notion of DNA methylation topology being considered as a potential indicator of causal impacts on chromatin distribution with a conceivable application in epigenetic drug toxicology.

## Background

DNA methylation is a crucial epigenetic modification of the human genome beyond the DNA sequence level that is involved in regulating many cellular processes [1]. Cancer cells frequently exhibit abnormally high levels of DNA methylation in gene-specific CpG-rich promoter regions [2-5]. Furthermore, DNA methylation also occurs at non-CpG islands within the major part of the genome known as heterochromatin [6,7], which plays a key role in nuclear architecture and genome stability [8-10]. It is now clear that DNA hypomethylation in human cancer is also very frequent and affects more cytosine residues than does DNA hypermethylation, accounting for a net loss of 5-methylcytosine (global DNA hypomethylation), as observed in many cancers [11-14]. The reversible nature of epigenetic imbalances in various types of cancers constitutes an attractive therapeutic target. The goal of epigenetic therapy in cancer is the reprogramming of aberrant cells towards normal phenotypes. In this regard, the drug discovery field has so far been mostly focusing on screening the effect of candidate agents on the levels of molecular cell signaling and metabolism. However, in recent years of the post-genomic era, chromatin conformation and the higher–order genome organization, which set the framework for the global orchestration as well the locus-specific regulation of gene expression in the human cell nucleus [15-18], are gaining more attention in therapy; the reason being that these functional structures can become affected as a consequence of epigenetic interference by chromatin-modifying agents such as inhibitors of DNA methylation [19].

Catalytic DNA methyltransferase (DNMT) inhibitors have been so far categorized into two classes: nucleoside analogues and non-nucleoside analogues [20]. The two nucleoside analogues, 5-azacytidine (AZA, Vidaza™) and 5-aza-2^′^-deoxycytidine (decitabine, Dacogen™) are the most advanced in their category, having received US Federal Drug Agency (FDA) approval for their use in treating myelodysplastic syndrome (MDS) and hematopoietic malignancies [21,22]. Zebularine (ZEB) or 1-β-D-ribofuranosyl-2(1H)-pyrimidone has recently emerged as a new DNMT inhibitor (DNMTi), with properties that makes it a potential drug candidate for oral administration: (i) stability at pH ranges between 1.0 and 7.0 in aqueous solutions, (ii) far less toxicity than AZA and decitabine to cultured cells, and (iii) no detectable toxicity in a T-cell lymphoma mouse model [23-27].

The specific mechanism of DNA methylation alterations induced by azacytidine nucleoside analogues is complex and not fully understood. Azacytidine is thought to form a stable covalent bond with DNMTs after its incorporation into genomic DNA, thereby trapping the enzyme and sequestering it from transferring methyl groups to other regions of the genome [28-30]. Such a passive mechanism of DNA demethylation as a result of exposure to DNMTi has been proposed and is thought to progress with several cell divisions, after which DNMT levels increase and specific gene regions show re-methylation. Azacytidine treatment of cells also was shown to induce degradation of DNMT1 via the ubiquitin-activating proteosomal pathway [31], as well as p53-mediated cell cycle arrest and DNA repair [32]. Chromatin packaging and organization are altered in cells treated with azacytidine. Nucleosome depletion of symmetrically demethylated gene loci have been demonstrated after drug treatment [33]. However, it should be noted that there are additional reports indicating that genomic regions with AZA DNA-DNMT adducts are improperly packaged and transcriptional activation can only occur with DNA repair and recruitment of other protein factors [34,35].

**Figure 1:**
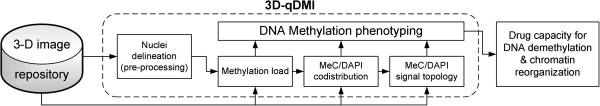
**Workflow of 3D quantitative DNA methylation imaging and analysis.** Image data acquired by high-resolution microscopy is subjected to a pre-processing step, in which cell nuclei (as areas of interest) are segmented, followed by DNA methylation phenotyping. This step comprises three modules, by which recorded signals in the MeC and DAPI channels are extracted for measuring: global MeC load, MeC/DAPI signal codistribution, and MeC and DAPI signal topology within the nuclear space. The retrieved information is used to assess the capacity of a drug for DNA demethylation and concurrent chromatin reorganization.

To date, differential DNA methylation analysis has been quantitatively performed mostly by means of molecular approaches including electrophoretic, chromatographic, PCR-based, array-based, and sequencing technologies [36,37]. Furthermore, evidences indicate that DNMTi also influence repressive histone marks leading to changes in nucleosome positioning [33,34]. Hence, a novel nucleosome footprinting assay was developed, which takes advantage of improvements in these technologies and focuses on the characterization of locus-specific as well as genome-wide chromatin conformation with respect to DNA methylation on a single molecule level [38,39]. Such an analytical tool can be used to characterize the differential chromatin states and changes thereof that can occur under drug influence and would benefit therapeutic design: as demethylating drugs may, in addition to their physiologic role, also affect chromatin architecture and related gene expression programs in cells [40-47]. The structure and function of the human genome are so intricately intertwined that understanding its regulation requires viewing the genome as a dynamic three-dimensional entity that emerges from iterations of dynamic folding of the primary chromatin structure, the so-called nucleosomal array: also considering the mass of heterochromatin that is largely repressed and condensed through DNA methylation and histone-tail modifications, which are perturbed in complex diseases [17,18]. The immunodeficiency, centromere instability and facial anomalies (ICF) syndrome is a classic example, in which normally highly compacted juxtacentromeric satellite DNA is found hypomethylated and decondensed in chromosomes 1 and 16 [48]. Therefore, the higher genome organization of DNA provides an additional layer of cell-specific information that could render itself valuable in the evaluation of drug action, as it has potential to be translated into high-throughput and cost-efficient pre-clinical genotoxicity assays [19]. In this sense, little is known about the spatial progression of DNA hypomethylation in cell nuclei in response to DNMTi. The analysis of global nuclear DNA methylation patterns could provide a useful means in assessing said epigenetic effect of this class and possibly other classes of drugs in a large number of single cells, as the underlying molecular processes may involve large-scale chromatin reorganization visible by light microscopy [40-44,49-51]. Recently introduced, 3D-qDMI, can measure DNA methylation changes *in situ*, through the differential analysis of relevant nuclear structures that are represented by methylated CpG-dinucleotides (MeC) and global DNA [40-42] (Figure 1). Our analyses revealed significant differences in image patterns of MeC and heterochromatin-derived signals between untreated AtT20 mouse pituitary tumor cells and a subpopulation of these cells treated with AZA, which has been reported to change DNA methylation patterns on a genomic scale [52]. Furthermore, the recently upgraded methodology was able to monitor the dual effect of demethylating agents in human cancer cells: (a) a global decrease in MeC content, and (b) the subsequent reorganization of highly compact heterochromatic regions of the genome as reflected by a significant decrease of DAPI intensity in the relevant nuclear areas. The effects resulted in LIM and LID sites, whose distributions can be mapped within cell nuclei [44]. This approach supports profiling at single-cell level, and provides a rapid display of cell-specific DNA methylation (MeC) phenotypes that is related to drug response in targeted cells. Initial results obtained with 3D-qDMI indicated towards the relatively gentle effect of zebularine on the genome, an observation that is concordant with reported studies based on molecular profiling. Initial proof-of-principle analyses focusing more on technology development were restricted to the application of one concentration per epigenetic drug. Here we report on the first-time probing of the 3D-qDMI system’s utility in a dose-dependent manner: by administration of a larger concentration range of the relatively more gentle nucleoside zebularine in comparison to its extensively characterized more aggressive analogue 5-azacytidine [23-27,53,54]. The notion was to follow a more gradual DNA demethylation effect of the agents on 5-methylcytosine topology, along with cytotoxicity evaluations, in the two *in vitro* models, DU145 prostate cancer cells and Huh-7 hepatocarcinoma cells, which have known sensitivity to both drugs [55-59].

## Methods

### Cell culture and drug treatment

DU145 human prostate cancer cells were obtained from American Tissue Culture Collection (catalog number HTB-81, ATCC). The vendor certifies authentication of cells using a variety of techniques such as short tandem repeat (STR) analysis and cytogenetic analyses (G-banding, fluorescence *in situ* hybridization). Huh-7 cells were a gift from Dr. Vaithilingaraja Arumugaswami (Cedars-Sinai Medical Center, Los Angeles, CA). The cells were propagated for less than six months after receipt and resuscitation. Cells were grown in Dulbecco’s modified Eagle’s medium (DMEM, Cellgro) supplemented with 10% newborn calf serum, and 1% antibiotic/antimycotic (1000 units/ml penicillin G sodium, 10 mg/ml streptomycin sulfate) (Gemini Bio-Products), in 5% CO_2_, 37°C. Cells were plated at 1 × 10^5^ cells onto coverslips in multi-well plates in replicates, and allowed to attach for 24 hours. For dose dependency assay, wells were divided into two groups: (i) control populations that were not treated for 72 hours, and (ii) populations of cells treated with two different drugs at different concentrations for 72 hours: 0.5 μM, 1 μM, 2.5 μM, 5 μM, 10 μM and 20 μM of 5-azacytidine (Sigma-Aldrich), and 8 μM, 40 μM, 200 μM, 500 μM and 1000 μM of zebularine (Sigma-Aldrich), all in DMEM. For all cells, drug concentrations were freshly prepared prior to administration, and the drug-medium mixture was changed every 24 hours. Subsequently, cells were partially fixed for immunofluorescence and partially harvested for cytotoxicity testing by flow cytometry.

### Cell synchronization

DU145 prostate cancer cells were arrested in G0/G1 and G2-phases following previously established protocols [60,61]. Briefly, cells were seeded onto glass coverslips at a concentration of 10^5^ cells/ml for immunofluorescence staining and subsequent imaging via confocal microscopy. A parallel set of cultures (at the same concentration) was maintained in culture flasks, for flow cytometry. All cells were first allowed to attach and grow for 24 hours in regular proliferative medium (DMEM/10% FBS/1% penicillin/1% streptomycin), which was then replaced by serum-deprived DMEM for 72 hours, followed by a recovery period of 4 hours, in which cells were maintained again in regular proliferative medium. G0/G1 populations were partially fixed at this point for use in either immunocytochemistry or FACS. The remainder cultures were processed for a double-thymidine block to enrich cells in G2-phase: (i) first blocking with deoxythymidine (Sigma) at 2 mM for 18 hours, (ii) recovery in regular proliferative medium for 12 hours to escape S-phase, (iii) second blocking with 2 mM deoxythymidine for another 18 hours, and (iv) second recovery in regular proliferative medium for 8 hours, to release cells into G2. At this point G2-cells were fixed for further experimentation. Enrichment efficiency was checked by propidium iodide (PI) staining of cells and nuclear DNA content analysis, following standard protocols as previously described in Wong et al. [62]: cells were fixed in 70% ethanol/PBS and maintained for at least 4 hours at 4°C; then incubated in 5 μg/ml PI (Sigma) for 30 minutes at 37°C immediately prior to flow cytometry with a FACScan (Becton Dickinson). FACS data were analyzed using the ModFit LT program (Verity Software House, Topsham, ME, USA).

### Cytotoxicity assay

Induction of apoptosis and cell viability was analyzed in cells that were treated as replicates in parallel to cells that were subsequently analyzed by immunofluorescence. For that purpose, 2×10^5^ cells/ml were stained with Annexin V (7-AAD) and PI, respectively [63]. In essence, trypsinated cells from parallel wells were processed with the Annexin V-FITC Apoptosis Detection Kit I (BD Biosciences). Cells (1×10^5^) were incubated for 15 minutes at room temperature with 7-AAD and PI in a total volume of 510 μl comprised of 5 μl of each of the fluorescent dyes, each and 500 μl of 1X binding buffer. Controls with unstained cells and cells stained with either dye alone were used for FACS setup. Samples were analyzed at emission wavelengths of 530 nm (for Annexin V-FITC) and 650 nm (for PI) using FACScan. The fluorescence of 10^4^ cells was acquired and analyzed with CellQuest software (Becton Dickinson).

### Immunofluorescence and image acquisition

In order to preserve the three-dimensional structure, cells cultured on glass coverslips in 12-well microplates (Costar, Corning) were fixed with 4% paraformaldehyde/phosphate buffered saline (PBS) (Sigma-Aldrich) and processed for immunofluorescence as previously described [64]. The following antibody sets were used: a monoclonal mouse anti-5-MeC antibody (Clone 33D3, Aviva Systems Biology, San Diego, CA) together with an Alexa488-conjugated polyclonal donkey anti-mouse IgG (H + L) (Invitrogen), and a polyclonal rabbit anti-H3K9me3 antibody (Active Motif) together with an Alexa647-conjugated chicken anti-rabbit IgG (H + L) (Invitrogen). All specimens were counterstained with DAPI. Specimens were imaged by a confocal laser-scanning microscope (TCS SP5 X Supercontinuum, Leica Microsystems Inc.) that allows for any excitation line within the continuous range of 470 to 670 nm, in 1 nm increments. The system was additionally equipped with a 405 nm diode laser line for excitation of DAPI fluorescence. Serial optical sections were collected at increments of 200–300 nm with a Plan-Apo 63X 1.3 glycerol immersion lens (pinhole size was 1.0 Airy unit). To avoid bleed-through, the imaging of each channel was performed sequentially. The typical image size was 2048 × 2048, with a respective voxel size of 116 nm × 116 nm × 230.5 nm (*x*, *y*, and *z* axes), and resolution was 12 bits per pixel in all channels. Fluorescence intensity of MeC-signals and DAPI-signals from optical two-dimensional sections were recorded into separate 3D channels. Raw images were obtained as Leica Image Format (lif) and offline-converted to a series of TIFFs for downstream image analysis.

### 3D image analysis

Image analysis was performed in three main steps, as comprehensively described in [43,44]: 1) image segmentation resulting in the delineation of a 3D shell for each individual nucleus; 2) extraction of MeC and DAPI signal intensity distributions within each 3D shell; 3) assessment of cell population heterogeneity through 2D histograms of MeC versus DAPI distribution patterns, utilizing K-L divergence, and 4) the mapping of LIMs and LIDs within individual nuclei. A newly added analytical component for this study was the calculation of mean intensity of MeC signals. Images in each two-channel 3D stack were acquired under nearly identical conditions and modality settings, and so the drift of the settings during acquisition is considered minimal and can be neglected. For codistribution analysis, the MeC and DAPI signals were mapped as respective 2D scatter plots, and following [43] the Kullback–Leibler (KL) divergences were calculated between individual 2D plots (nuclei) and the reference 2D plot (cumulative plot from all nuclei in one drug/concentration experiment). Based on the KL value, cells were categorized as: *similar KL*_*G*_ ∈ [0, 0.5), *likely similar KL*_*G*_ ∈ [0.5, 2), *unlikely similar KL*_*G*_ ∈ [2, 4.5), and *dissimilar KL*_*G*_ ∈ [4.5, *∞*) in order to evaluate a ratio of similar and dissimilar cells. For localization of resulting LIM and LID sites, the nuclei were analyzed by an algorithm introduced in [44]. Briefly, segmented nuclei were eroded at a constant voxel rate of 1.32 μm × 1.3 μm × 0.25 μm, and MeC and DAPI signals were recorded as integrated intensity values within each nuclear shell. Then, local densities of LIM and LID sites as well as LIM and LID profiles were determined for each nuclear shell as the subset of voxels within a defined intensity range between two thresholds measured separately for each channel (MeC and DAPI): t_bcg_ is the threshold value for the background, and t_Q_, which separates high-amplitude from low-amplitude intensities, as explicitly described in [44]. All analytical findings related to image processing including numerical results, MeC/DAPI codistribution patterns, individual and combined MeC/DAPI images, LIM/LID outputs of cells were exported by means of a graphical user interface to text or graphics files respectively for further statistical analyzes. A built-in pseudo-coloring of KL divergence, and LIM and LID site shading was superimposed onto original images to facilitate visual reading and evaluation of experimental data.

### Antibody specificity and sensitivity test

**Figure 2:**
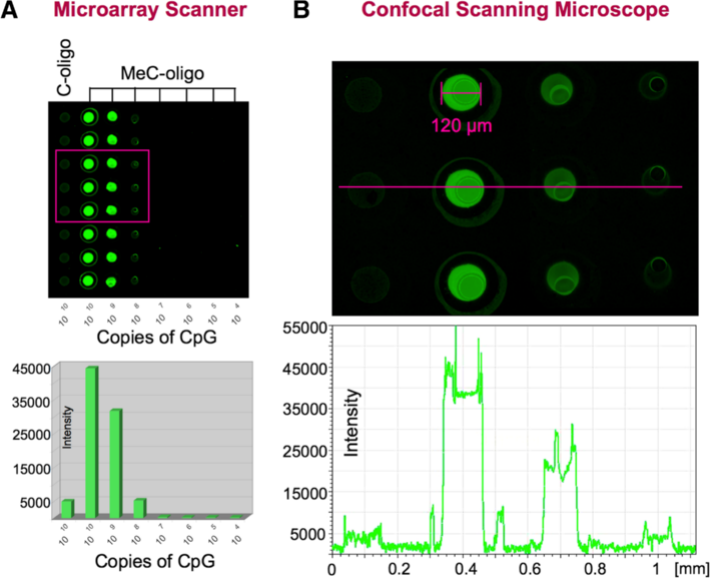
**Specificity and sensitivity of used anti-methylcytosine antibody.** (**A**) The antibody properties were assessed by an indirect immunofluorescence assay, in which the monoclonal anti-MeC antibody for this study — used at the concentration of 1 μg/ml in combination with a secondary antibody (Cy3-conjugated anti-mouse IgG1 at 5 μg/ml), i.e. at the same concentrations as in the cellular assay — was hybridized to a spotted array with two types of short 24-mer oligonucleotides immobilized onto a glass slide: C-oligo that included two CG dinucleotides and its methylated counterpart, the MeC-oligo printed at various dilutions that correlate with different approximate CpG copy numbers (10^10^–10^4^) . Each DNA probe was spotted as octuple. The specific antibody, detected with a microarray scanner at 5 microns resolution, shows best signal-to-noise (background and non-specific binding to unmethylated C-oligo) ratio at a copy number of 10^10^. The signal (false-colored in green) decreases in a CpG copy number-dependent manner. (**B**) Similar average intensities were obtained, when a sub-area (magenta box in Figure 2A) of the same array was subjected to confocal scanning microscopy at 200 nm horizontal resolution. The line scan (magenta) shows the more detailed intensity profile across the four different types of spots and the intermediate gaps (coated glass slide/background).

The specificity and sensitivity of the applied anti-5-methylcytosine antibody used in this study was assessed with a test-microarray as shown in Figure 2. Antibody testing was performed by an immunofluorescence assay utilizing a custom made spotted microarray (Full Moon Biosystems) comprising multiple copies of two synthesized 24-mer oligonucleotide probes that were immobilized onto glass microscopic slides: 5^′^-TCGTTTTTTTTTTTTTTTTTTCGT-3^′^ (C-oligo) (MWG Biotech), and its counterpart 5^′^-T^me^CGTTTTTTTTTTTTTTTTTT^me^CGT-3^′^ (MeC-oligo) (Biopolymers-Thermo Scientific), in which the two cytosine molecules were replaced by methylcytosine. Immunofluorescence was performed with the primary anti-methylcytosine antibody and the Alexa488 conjugated secondary antibody, and alternatively a Cy3-conjugated goat anti-mouse IgG1, using a denaturing step with hydrochloric acid, a blocking step with 3% BSA in PBS, and stringency washes as described for the *in situ* immunofluorescence assay above. Fluorescence detection was performed comparatively at 5 microns resolution with a microarray scanner (G2565BA, Agilent Technologies) equipped with a helium-neon laser (633 nm) to excite Cy3, and the above-mentioned confocal microscope with a Plan-Apo 10X 0.7 lens.

### MethyLight assay for repetitive elements

MethyLight assays for measuring DNA methylation content of Alu, Satα and Sat2 repeat sequences were performed as previously described by Weisenberger et al. [65]. Briefly, genomic DNA was extracted from harvested Huh-7 cells and 1 μg of genomic DNA was converted with bisulfite and recovered using the Zymo EZ DNA methylation kit (Zymo Research, Irvine, CA), as recommended by the manufacturer. Aliquots of the bisulfite-converted DNAs were used in separate MethyLight assays as previously described [65]. MethyLight data specific for the three types of repetitive elements were expressed as percent of methylated reference (PMR).

## Results

### Zebularine exerts a comparably lower degree of cytotoxicity than 5-azacytidine

We evaluated cultured DU145 prostate cancer cells and Huh-7 hepatocellular carcinoma cell lines for imaging-based DNA methylation analysis using the 3D-qDMI system to determine DNA methylation phenotypes of cells after 5-zacytidine and zebularine administration. These drugs have been used with a variety of cancer cell lines, including DU145 and Huh-7 cells, and described as being compatible to a large extent with cell viability and cell division [25,40,53-59]. The azanucleoside drug concentrations applied here were in the range as previously reported by investigations utilizing molecular nucleic acids-based assays.

**Figure 3:**
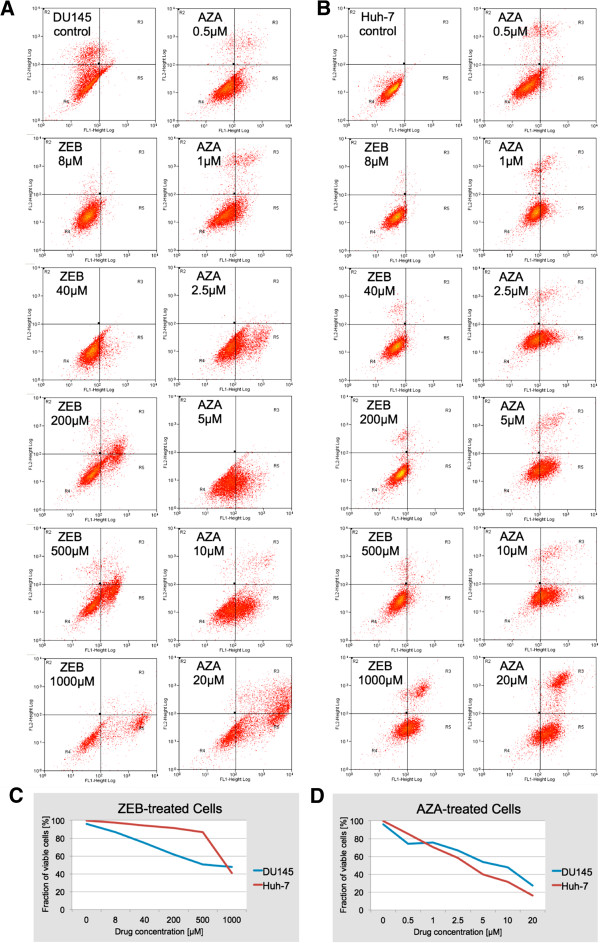
**Cytotoxicity of agents analyzed by FACS.** Results for the comparative analysis of the effects of zebularine (**A**) and 5-azacytidine (**B**). FL1-Height and FL2-Height represent Annexin V-staining and PI staining, respectively. Untreated control cell populations of DU145 and Huh-7 cells consist of a major portion of viable cells (> 90%). This reference profile changes with treatment of cells with different drug concentrations applied for 72 hrs. The viability of cells was normalized against the viability in the control population (considered as 100%) and displayed as a chart for zebularine (**C**) and 5-azacytidine (**D**).

For cytotoxicity analysis, we tested cells that were cultured in parallel to those used for imaging-based DNA methylation analysis. Cytotoxicity analysis was divided into an initial cell counting with an aliquot of trypsinized cells, followed by staining of the remainder of the cells with Annexin V and propidium iodide, and subsequent flow cytometry. Zebularine was administered at molar concentrations (8–1000 μM) that were one to two orders of magnitude higher than AZA (0.5–20 μM) with comparable cytotoxic effects (Figure 3A, 3B). Therefore, ZEB can be categorized as an agent with a much lower cytotoxic potential. This has also been described in previous reports [23,27]. In more detail, flow cytometry revealed a higher sensitivity of DU145 for ZEB compared to Huh-7 cells: IC_10_ and IC_50_ of ZEB were 8 μM and 500 μM, respectively for DU145 versus 200 μM and 1000 μM for Huh-7. In the case of AZA we experienced fewer discrepancies: IC_10_ was 0.5 μM for both cell types and IC_50_ was measured at 10 μM for DU145 and 5 μM for Huh-7. A greater than two-fold increase of the apoptotic fraction (Annexin V-positive) for AZA-treated cells of both types was detected at 2.5 μM, and for ZEB-treated DU145 cells at 200 μM, whereas same effects were registered in Huh-7 cells at 1000 μM (Figures 3C and 3D). For the comparative analysis of the two drugs at different concentrations, 2.5 × 10^5^ cells were initially seeded onto coverslips. After 72 hours we recorded a tripling of naïve cells and only a doubling for both cell types at the drugs’ IC_10_ levels. Analogously, at IC_50_ ZEB-treated cells did not show any population growth, whereas AZA-treated cells showed significant reduction of their populations: Huh-7 cells were reduced to 50% and DU145 cells even to 10% of their original confluency. The results underline the ability of ZEB to reduce proliferation at higher doses without acting discernibly cytotoxic as demonstrated by AZA.

### High variation in DNA demethylation and differential drug sensitivity revealed by cell-by-cell imaging

**Figure 4:**
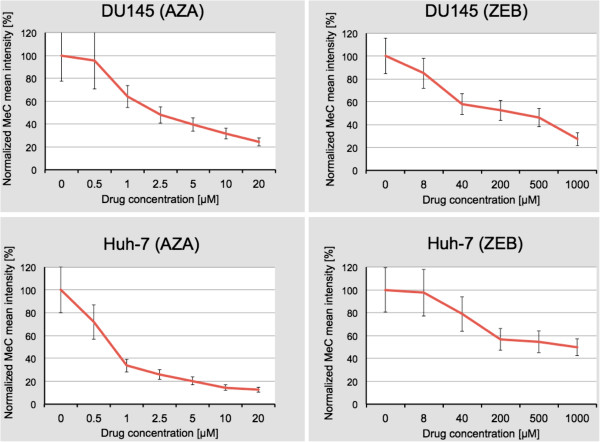
**Normalized MeC mean intensity in untreated and drug-treated cells.** Decline of signal intensity is plotted as a function of drug concentration. Standard deviation of *I*_MeC_ for untreated cells and cells treated with lower drug concentration is significantly greater than for cells treated with higher drug concentration. Comparative reduction in overall DNA methylation (as percentage drop compared to untreated controls) can be inferred for both drugs in DU145 cells, although at concentrations of one to two magnitudes lower for AZA than for ZEB. Huh-7 cells show comparatively less loss of *I*_MeC_ at highest zebularine concentration (1000 μM).

Untreated cells as well as cells treated separately with AZA and ZEB were automatically imaged from different areas of each coverslip. Imaged sub-populations were batch-processed off-line using 3D-qDMI software. We evaluated drug action by measuring two parameters on a per-cell basis: (i) the 5-methylcytosine load of nuclei, which we refer to as the mean intensity of the MeC signal (*I*_MeC_), and (ii) the nuclear topology of the MeC versus DAPI signals. The number of cells that we could extract the MeC-specific signals from depended on the cytotoxicity level of the drugs: resulting in a certain density of intact cells for each drug type, and subsequently the number of analyzable nuclei per image frame. We determined *I*_MeC_ across all resulting nuclei for each drug type. Figure 4 illustrates relevant statistics in naïve cells and each of the treated populations. The mean intensity was evaluated by a two-sample Kolmogorov-Smirnov test run for the experiments with each combination of drug and cell line. In DU145 (ZEB) cells, a significant difference was observed between all distributions of *I*_MeC,_ except for the 200 μM dose that was not significantly different from 40 μM and 500 μM. In DU145, cells treated with AZA the distributions of *I*_MeC_ for untreated and 0.5 μM were not significantly different. Also, no significant difference was observed between 10 μM and 20 μM in Huh-7 (AZA) cells, as well as between untreated and 8 μM dose, and the three highest concentrations in Huh-7 (ZEB) cells. The significance level in each test (β) was determined by Bonferroni correction (β = α/n) for α = 0.05, n = 6 or 7 for ZEB and AZA treatments, respectively.

The experimental results confirm the hypomethylating effect of both drugs; the increase of drug concentration causes a progressive loss of globally measured MeC-specific signal in nuclei (*I*_MeC_) and a decrease of *I*_MeC_ spread (Figure 4). Interestingly, AZA, at the highest concentration applied (20 μM), reduced the *I*_MeC_ stronger in Huh-7 cells (88%) than in DU145 cells (75%), whereas ZEB at the highest concentration (1000 μM) reduced *I*_MeC_ in DU145 cells at 72% versus 50% in Huh-7 cells, on average. However, when comparing global DNA methylation of cell nuclei at the equitoxic levels, ZEB showed a much stronger DNA hypomethylation effect than its nucleoside analogue at IC_10_ — 15% versus 5% for DU145 and 43% versus 18% for Huh-7 — then a milder effect at IC_50_: 54% versus 69% for DU145 and 50% versus 80% for Huh-7 cells. These results are in agreement with previous studies [24,26,54], and underline the less toxic effect of zebularine on cells and the milder nature of the drug when compared to AZA. In other words, AZA-treatment in both cell lines showed an approximate reduction of *I*_MeC_ at 63% between IC_10_ and IC_50_ concentrations, whereas the *I*_MeC_ reduction leap was significantly different between ZEB-treated cells: *I*_MeC_ ≈ 39% for DU145 and only 7% for Huh-7 cells. The data indicate that if DNA hypomethylation effects would be influencing cytotoxicity, dose–response may vary for different drugs in different cells.

### Dose-dependent topological progression of DNA hypomethylation correlates with cytotoxicity

**Figure 5:**
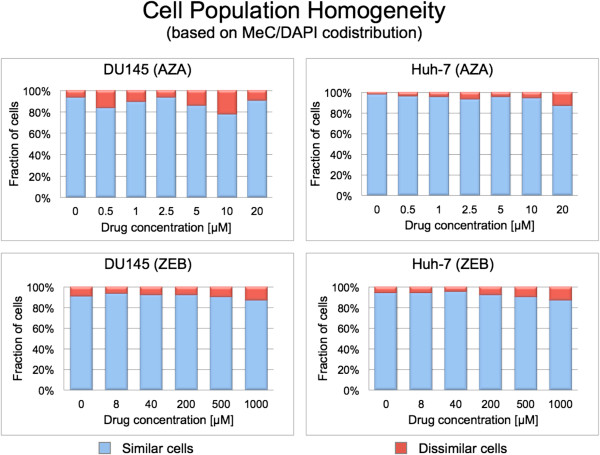
**Cell population homogeneity measurement.** Left diagram (different drugs): normalized proportions of the different cell populations (n ≈ 300 is the number of cells analyzed for each category); untreated control cells show a high fraction (≥ 95%) of pooled similar cells (*KL* < 4.5); this is true also for the majority of drug concentrations with both cell types (similarity on average ≥ 90%); the lowest homogeneity values being still relatively high at 82% for DU145/0.5 μM AZA and 79% for DU145/10 μM AZA. For simplification purposes, the rest of the cells were summarized as *dissimilar* cells (*KL* ≥ 4.5) in this display.

**Figure 6:**
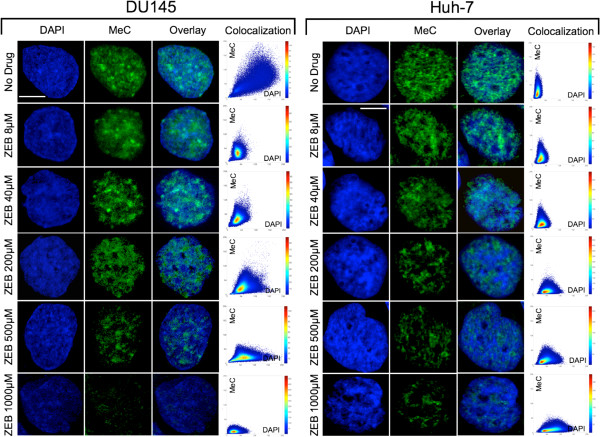
**Global differential DNA methylation phenotypes in response to zebularine concentration.** Maximum intensity projections (MIP) of 3D-imaged nuclei of DU145 cells with high similarity values within their population category are selected for display (bars are 5 μm). Gradual increase in reduction of MeC sites (false-colored green) was observed in correlation with an increase in zebularine concentration, similarly in both cell types. At lower concentrations (8 and 40 μM) global demethylation seems to be preferentially stronger at the nuclear periphery (delineated by DAPI, false-colored blue) and less DAPI-dense areas (supposedly euchromatin), and gradually affects more interior regions with increased drug concentrations. At higher drug doses (200 μM and especially 500–1000 μM), also more DAPI-dense areas (heterochromatin) have been demethylated. This latter effect is even more pronounced in the AZA-treated cell nuclei (data not shown in here). However, the majority of heterochromatic regions seem to retain their compact conformation. The respective scatter plots of the nuclei provide more quantitative information regarding changes in the MeC/DAPI codistribution as a consequence of drug application, especially for the lower drug doses: a demethylation of non-heterochromatic sites (MeC-positive, low-DAPI signals) is indicated for 40 μM compared to 8 μM, as judged by the decline of the graph slope. This trend correlates with increasing drug concentration. At 200–1000 μM the leveling of the slope towards the x-axis becomes obvious; additionally also strong DAPI-positive sites (heterochromatin) have started to become hypomethylated.

The analysis of the MeC/DAPI codistribution showed a high fraction of cells with pooled all *similar* categories in response to both drugs for all concentrations (Figure 4). ZEB-treated populations contained ≥ 90% similar cells, compared with AZA-treated populations with an average ≥ 85% and a slight tendency to drop for DU145 cells at 0.5 μM and 10 μM (82% and 79%, respectively). The cell population heterogeneity analysis was performed with an average total cell number of n = 300. Figure 5 displays normalized proportions of the two resultant categories of cells, and example MeC/DAPI codistributions are presented in Figure 6.

The effect of the drugs can be perceived as a reduction in the MeC signal, with a similar effect in both cell systems, when compared to nuclei of untreated cells. In case of each drug, we observed a dose-dependent reduction of the MeC-specific signal. At lower drug concentrations (ZEB: 8 μM and 40 μM; AZA: 0.5 μM and 1 μM) the nucleus still shows significant DNA methylation in its periphery, which becomes hypomethylated at medium to higher drug doses (ZEB: 200–1000 μM, AZA: 5–20 μM). This is accompanied by a decrease of DNA methylation at interior nuclear regions, gradually affecting also DAPI-dense areas that are attributed to heterochromatin. Zebularine at the 1000 μM dose shows extremely strong hypomethylation in the entire nuclear space, including a large portion of heterochromatin in the nuclear interior (Figure 5). In comparison, AZA shows similar effects already at 5 μM (data not shown). These observations support our findings presented in Figure 3. The visual impressions of DNA hypomethylation in response to drug type and concentration were confirmed by quantitation of MeC and DAPI signal codistributions in the respective nuclei and displayed as accompanying scatter plots (Figure 6).

**Figure 7:**
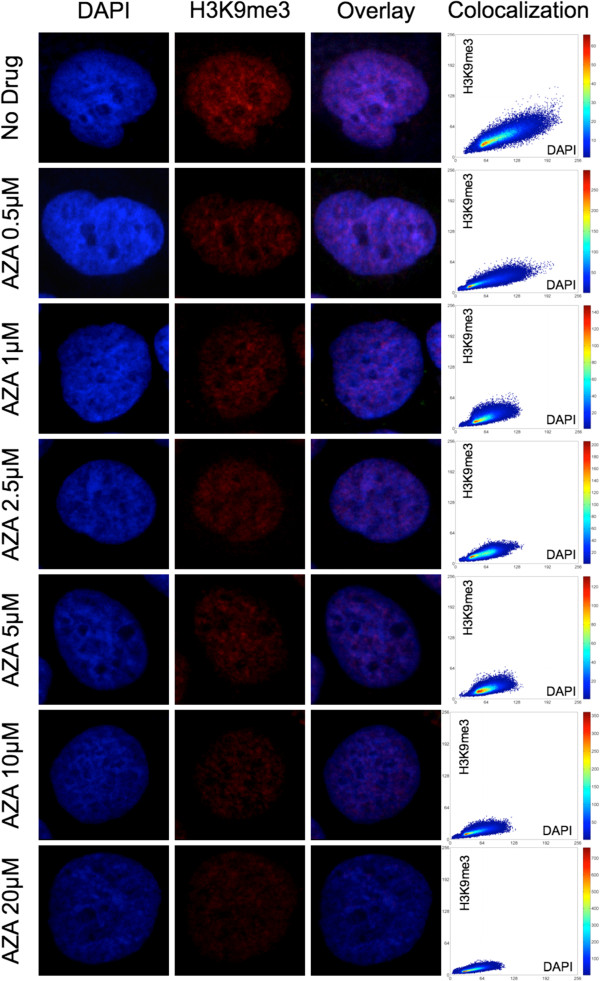
**Nuclear codistribution of H3K9me3 and global DNA.** Heterchromatin-associated H3K9me3 (red) significantly colocalizes with DAPI-intense areas (blue) in untreated and AZA-treated Huh-7 cells. The scatter plots show that the signal in both channels gradually decreases with increasing drug concentration. The relatively stable inclination of the colocalization graph indicates that both signals regress proportionally, which could be interpreted as a tight correlation between heterochromatin and DAPI-intense regions.

In addition to AZA-treatments, a subset of Huh-7 cells was separately stained for covisualizing differential spatial distribution of histone H3 lysine 9 trimethylation (H3K9me3) and global DNA. H3K9me3 is associated with heterochromatin and is involved in the recruitment and binding of heterochromatin protein 1 (HP1), with subsequent chromatin condensation and compaction [66,67]. Therefore, we monitored this marker in sample cells to specifically record changes in higher-order heterochromatin organization in conjunction with AZA drug application (Figure 7). Our findings show a high degree of colocalization between the H3K9me3 and DAPI signals in untreated cells and cells treated with the entire spectrum of applied AZA concentrations. Therefore, one can assume that DAPI signals could be utilized as a surrogate marker for visualizing changes of global heterochromatin organization. Furthermore, it is conceivable that a reduction in H3K9me3 could lead to local DNA decondensation as extensively reported elsewhere [10,68]. These findings support our topologic approach in using DAPI signals as a convenient way of reporting changes in heterochromatin organization and distribution, extensively discussed in previous works [43,44]: as we found that DAPI staining is compatible with the hydrochloric acid-treatment conditions of fixed cells we applied for MeC-signal retrieval without any detectable obscuring of both signals [64].

**Figure 8:**
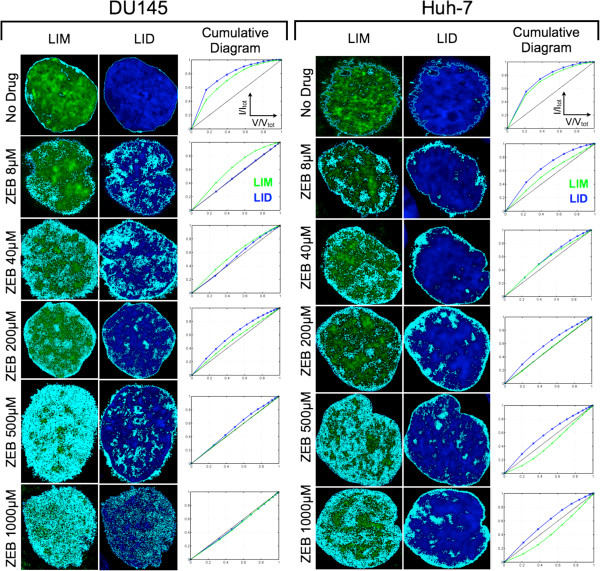
**Differential LIM and LID topology in zebularine-treated cells.** LIM and LID sites detected in the range of (t_bcg_, t_Q_) in individual cells from Figure 6 (marked in cyan color) are superimposed onto an intermediate optical section in the respective MeC (green) and DAPI (blue) channels (left and middle columns). LIM/LID density curves obtained through morphological erosion of nuclei are shown as cumulative diagrams (right column). These sites correspond with codistribution patterns in Figure 4 for the respective nuclei. The number of graph points in the third column is associated with the number of detected shells; the value of each point refers to the fraction of all sites found in the nucleus up to the next shell. V is the shell volume and V_tot_ is the total volume of a nucleus. The argument V/V_tot_ = 0.5 distinguishes all LIM sites that are localized in the peripheral half of the nucleus from LIMs of the interior half (V/V_tot_ > 0.5). The diagonal line across each plot in represents hypothetically equal density of LIM or LID sites across the nuclear volume. Similar degrees of high rim-like LIM and LID densities are apparent in untreated prostate and hepatic cancer cell nuclei. LIM sites quasi-linearly expand towards the nuclear interior upon the increase of zebularine concentration, with ZEB at 500–1000 μM show large coverage in Huh-7 nuclei and nearly full coverage throughout DU145 nuclei. Also LIDs show an increased distribution in treated cells versus naïve cells, but the changes are more similar across all the applied ZEB and AZA concentrations in both cell types, with most increases occurring in the exterior shells of the nuclei.

**Figure 9:**
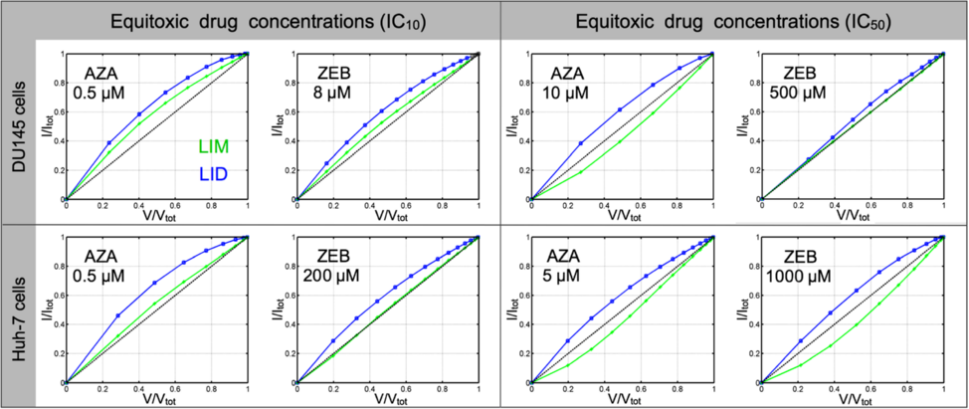
**Low-intesity MeC and low-intesity DAPI site distribution for equitoxic drug concentrations.** For the two cell lines, the responding distributions of LIM and LID sites are quite similar between equitoxic drug concentrations: LID distributions did not notably vary between the two concentrations as much as LIM distributions did between IC_10_ and IC_50_ concentrations. However, a slight difference in LIM distribution was measured for DU145 at IC_50_: at 500 μM LIM distribution seemed more similar to distribution at IC_10_.

To further emboss the differential spatial distribution of global DNA and its methylated portion, we focused on the changes in the localization of LIMs and LIDs, as subsets of nuclear signals that represent hypomethylated sites and areas of lower DNA density in naïve and treated cells. As illustrated in Figure 8, both LIM and LID sites in the untreated cells have a rim-like localization at or close to the nuclear border (*LIM*_0.5_ = 0.80–0.85, and *LID*_0.5_ = 0.85) for both cell types after zebularine treatment, while only 15–20% of LIMs were located in nuclei. In cells comparatively treated with ZEB and AZA, the nuclei showed an increased portion of interior LIM and LID sites after treatment with each drug. The increase in LIM sites is correlated with the increase in ZEB concentration: on average the LIM-portion in DU145/Huh-7 is raised to ~30%/~40% at 8 μM, ~40%/~40% at 40 μM, ~45%/~50% at 200 μM, 50%/60% at 500 μM, and ~55%/~65% at 1000 μM, respectively. In comparison, the LID-portion in the nuclear interior significantly expanded at lower ZEB concentrations: up to 50% at 8 μM for DU145 and 40% at 40 μM for Huh-7, but did not significantly change beyond this concentration in either cell type. In AZA-treated cells the change for LIDs was very similar, however, LIM sites increased up to 60% on average as can be inferred from the subset of data displayed for the equitoxic drug concentrations in Figure 9. For the two drugs, the responding distributions of LIM and LID sites are quite similar between equitoxic concentrations with a slight difference of IC_50_ for DU145 cells. Interestingly in this context, LID distributions did not vary substantially compared to LIM distributions between IC_10_ and IC_50_ concentrations. From these results we glean that an increase of global DNA hypomethylation can be traced in a dose-dependent manner. However, a significant concurrent reorganization of the genome based on changes in DAPI densities occurs already at the lower applied drug concentrations, and does not seem to become stronger at concentrations that are 25–100-fold higher. Therefore, the differential LIM and LID topology supplements the MeC/DAPI codistribution findings described in Figure 6. The respective diagrams of the cells show a flattening of MeC/DAPI codistribution and the increase of LIM sites concurrent with increasing dosage. Stronger hypomethylating effects at higher concentrations of AZA or ZEB were not accompanied by an additional increase of LID sites. Also, the increase in LIM distribution towards higher LIM densities reflects the spatial progression of DNA hypomethylation, which seems to positively correlate with drug-based cytotoxicity.

### MeC/DAPI codistribution patterns are independent from cell cycle interphases

**Figure 10:**
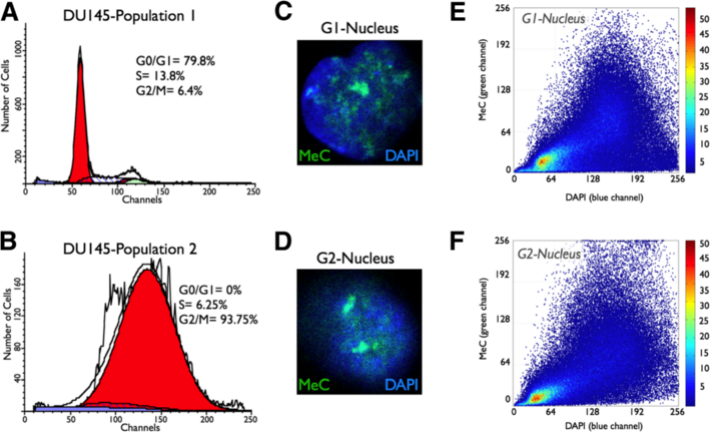
**Cell cycle-specific MeC/DAPI codistribution patterns.** Flow cytometry results show DU145 cell populations were efficiently arrested in G0/G1-phase (**A**) and enriched in G2-phase (**B**). The culture conditions were chosen to skip an enrichment of the cells in S-Phase. G1-cells and G2-cells from parallel populations were subjected to immunofluorescence and confocal imaging. The prototypic nuclei (**C** and **D**) of the two cell cycle phases (with a low KL-value) show very similar MeC (green) and DAPI/gDNA (blue) codistribution patterns, also confirmed by their respective scatter plots (**E** and **F**).

Interphase cells are largely divided into two prominent groups based on their cell cycle stage: G0/G1-phase and G2-phase, differing in DNA content. Compared to haploid G1-cells diploid G2-cells normally contain two copies of the genome after having undergone the intermediate S-phase, in which DNA is replicated. Therefore, we investigated the possibility of existing differences in MeC/DAPI distribution patterns between these two cell cycle phases. DU145 cells were synchronized in culture and arrested in G0/G1 and G2-phases. Cell stage-enriched populations were processed for immunofluorescence and 3-D imaging. We found that synchronized cell populations were comprised of an absolute majority of cells in interphase, as most of the barely attached and round metaphase cells are usually lost during the early synchronization steps (Figures 10A and 10B). Utilizing 3D-qDMI, we did not detect any significant differences for MeC/DAPI codistribution patterns between the two major cell cycle phases. Sample signatures of selected (*similar*) G1 and G2-cells with a low KL-value that represent typical global nuclear MeC phenotypes are shown in Figure 10 (C–F), and demonstrate similar codistribution patterns seen for untreated DU145 cells (Figure 5). Based on these results, we conclude that significant changes in MeC/DAPI patterns detected by 3D-qDMI are a result of drug action and not influenced by eventual cell cycle phase variability.

**Figure 11:**
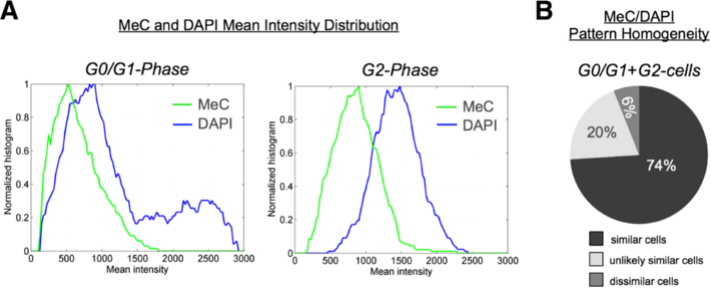
**Variability of MeC and DAPI intensities in synchronized cells.** (**A**) Mean intensities (normalized for n = ~200 cells for each) of global methylcytosine (MeC) and overall DNA (DAPI) nearly doubled between G0/G1-phase and G2-phase, with a large spread in MeC and DAPI signal distributions indicating high signal variabilities in synchronized cells. (**B**) In comparison MeC/DAPI codistribution patterns in the combined data of the same cells from the two phases exposed a high degree of homogeneity, which is a sign for high similarity in MeC phenotypes between cells of the two phases.

In comparison, when analyzing DNA methylation and DAPI loads of nuclei in synchronized cell populations, we found that the amplitude of the respective mean intensities *I*_MeC_ and *I*_DAPI_ has nearly doubled in G2 versus G0/G1 phase. However, the distribution of these two values shows a large spread in both phases (Figure 11). This fact demonstrates that although we could measure general load trends that most probably correlate with the doubling of the genome between G1 and G2 phases, overall mean intensities of global DNA and total MeC content can drastically vary, even between synchronized cells; therefore making it difficult to distinguish between their natural variation and strictly drug-induced changes. On the contrary, when MeC/DAPI codistribution data of the same G1 and G2 arrested cells were combined, the computationally merged population presented a high degree of homogeneity, as calculated by KL-divergence measurement. This confirms the high similarity between the MeC phenotypes of cells from the two different populations, and emphasizes on the robustness of MeC/DAPI patterns in evaluating drug-induced effects on nuclear DNA methylation topology.

### Analysis of DNA methylation levels in repeat sequences correlates with imaging results

**Figure 12:**
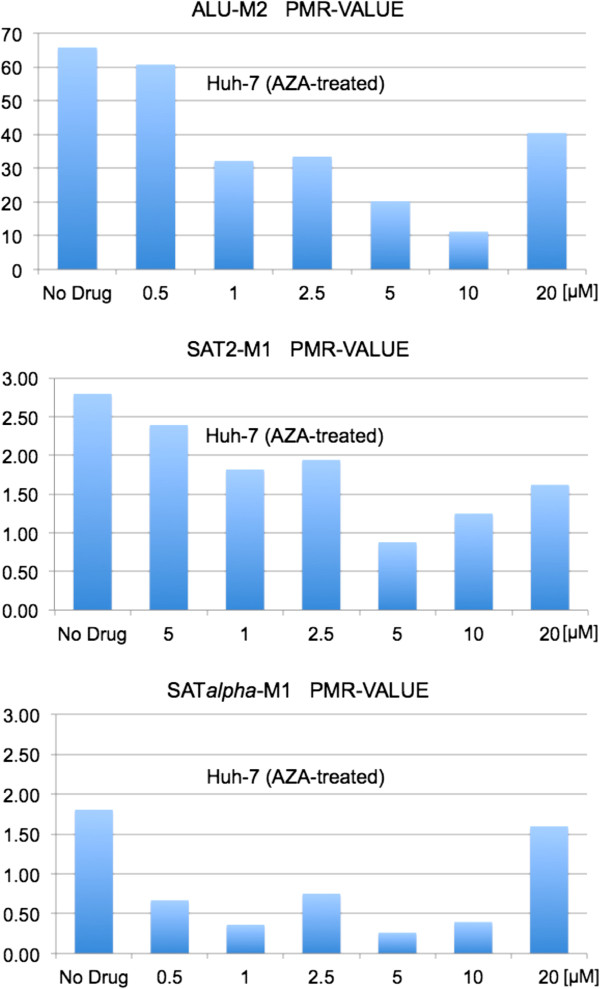
**Drug-induced changes in DNA methylation levels of repetitive elements.** DNA methylation levels of the three classes of repeat sequences Alu, Satα, and Sat2, assessed by specific MethyLight assays, significantly decreased in Huh-7 cells upon treatment with AZA. The degree of hypomethylation correlated with drug concentration, with the exception of an increase in DNA methylation seen for all three repetitive elements at the 20 μM AZA concentration.

For comparative analysis of differential DNA methylation loads and to verify the quantitative accuracy of 3D-qDMI, Huh-7 cells were subjected to AZA treatment under the same conditions (concentrations and exposure times) as for cells interrogated by image and flow cytometry, and analyzed using MethyLight technology, a real-time PCR based DNA methylation assay [65]. MethyLight assays measuring DNA methylation of repetitive element sequences have been previously described as accurate surrogates for quantitating global DNA methylation levels. Using this technique, we measured DNA methylation levels in the three of the most prevalent and highly methylated human repeat sequences: the short interspersed nuclear element (SINE) Alu sequences that are highly abundant in the human genome, as well as the pericentromeric Sat2 and the centromeric Satα, which both belong to constitutive heterochromatin. The choice of said targets was based on the facts that DNA hypomethylation of these sequences can lead to local chromatin decondensation and genomic instability, which have been well characterized in diverse cancers and other types of complex traits such as ICF syndrome [8,13,48]. Also, these repetitive elements have been shown to become hypomethylated after exposure to DNMTi [12-14,69]. The molecular assay revealed that DNA methylation levels in all three classes of repetitive elements showed similar trends and were in strong agreement with results observed for global DNA methylation with 3D-qDMI: the untreated cells record the highest level of MeC content with a gradual decline as the drug concentration increases, and a re-increase of DNA methylation for the 20 μM AZA dose. We believe that because of the purportedly extensive damage to cell integrity at the 20 μM AZA concentration, the more methylated (possibly drug-resistant) cells may have selectively survived (Figure 12). This was observed with microscopic imaging, in which the cell populations were significantly reduced compared to lower drug doses and contained larger numbers of highly methylated cells, which were excluded as outliers in 3D-qDMI analysis.

In order to draw direct comparisons between image-derived data and molecular sequenced-based results a correlation coefficient was calculated between *in situ* global DNA methylation levels, i.e. normalized MeC mean intensities of analyzed Huh-7 cells (obtained by 3D-qDMI, Figure 4) and DNA methylation levels measured (normalized PMR values) for each class of repeat sequence across AZA concentrations up to the 10 μM dose, as shown in Table 1. The comparison resulted in high correlations between the outcome of the two platforms, the highest being for the interspersed Alu sequences (R = 0.96), followed by pericentromeric Sat2 and centromeric Satα (R = 0.89 and 0.86, respectively).

**Table 1:** Correlation between imaging and sequence-based methylcytosine levels

	**3D-qDMI**	**MethyLight**		
**AZA concentration**	**Normalized MeC intensity**	**Normalized Alu PMR value**	**Normalized Sat2 PMR value**	**Normalized Satα PMR value**
No Drug	1.00	1.00	1.00	1.00
0.5 μM	0.72	0.92	0.86	0.37
1 μM	0.34	0.49	0.65	0.20
2.5 μM	0.26	0.51	0.69	0.42
5 μM	0.20	0.31	0.31	0.15
10 μM	0.14	0.17	0.45	0.22
20 μM	0.13	0.61	0.58	0.88
Correlation coefficient R*		0.96	0.89	0.86

* Correlation coefficient R was calculated between the normalized MeC intensities measured by 3D-qDMI and normalized PMR values of the three classes of repetitive elements obtained by MethyLight, excluding the values for 20 μM of AZA.

## Discussion

Epigenetic drugs including DNA methyltransferase inhibitors, which are meant to correct for DNA methylation imbalances in cells, constitute promising therapeutic approaches in the battle against cancer. The FDA-approved azanucleotides 5-azacytidine and decitabine are already administered to patients with hematologic neoplasias. Zebularine has emerged as a new member of this type of agents that has shown potentials for long-term oral applications, as a result of systematic comparative analyses [23-27,70,71]. However, most of the assessments have been performed utilizing molecular methods that reveal precise information regarding CpG methylation profiles of non-repetitive sequences, but are currently costly and time-consuming, if not challenged, when applied in a cell-by-cell mode. Nevertheless, we believe that analysis of cultured cell models *at single-cell resolution* is necessary to obtain a more global and cell systemic picture of drug action and efficacy in the search for new drugs as well as the epigenetic evaluation of existing drugs. Thus, high-content and high-throughput analyses, which have been supported by recent advancements in imaging technology and computational capacities, offer valuable means for rapid and cost-effective cellular phenotyping in drug screening [72]. Furthermore, the vast majority of studies so far have been focusing on assessing the hypomethylating potential of drugs on selected gene promoters in combination with cell viability testing for drug cytotoxicity and genotoxicity. However, hypomethylating agents can also perturb the epigenetic regulation of chromatin conformation, thus having an impact on the higher-order genome organization and nuclear architecture that regulate genome integrity and gene expression [19]. We were interested in tracking the progression and extent of such global structural changes, also in correlation with drug cytotoxicity to additionally elaborate on the verification of the 3D-qDMI system’s utility for the therapeutic field. Towards this end, we have conducted a comparative cell-by-cell evaluation of zebularine and its extensively characterized isoform 5-azacytidine based on their effects on global nuclear DNA and its higher-order organization in the cell nucleus. For the purpose of generating comparable topological data, we chose human cell culture models that have rendered themselves as sensitive to both agents, as well as cell culture conditions and drug doses that have been used previously in comprehensive studies to explore differential changes on the level of DNA methylation for targeted single-copy CpG sites. Our study includes standard viability testing for measuring cytotoxicity and upgraded 3D-qDMI for evaluating the demethylation effects on two levels: (i) changes in the load of nuclear MeC (*I*_MeC_), and (ii) alterations in the spatial codistribution of MeC and global DNA, including condensed heterochromatin regions that are represented by bright DAPI areas in the nuclei of cells. Our cytotoxicity data as well as the results of our topologic approach are strongly concordant with data presented by other investigators [23,25,26,73-75]. Drug response efficacy, as judged by the degree of spatial nuclear MeC/DAPI patterns, was comparably high for the two drugs across all concentrations.

In terms of cytotoxicity, we found that the Huh-7 hepatocarcinoma cells reacted more sensitively to zebularine than the prostate cancer cells. Nevertheless, for both cell types, zebularine elicited similar cytotoxicity levels at doses that were one to two orders of magnitude higher than for 5-azacytidine, thus can be considered as much less cytotoxic at near-equimolar concentrations. The results are in accordance with data from other investigations that have probed the two agents in various other cancer cell models such as bladder (T24), colon (HCT-116), ovarian (A2780 and HEY) and breast (MBA-MD-231 and MCF-7) cancer cell lines, as well as in acute myeloid leukemia cells (AML 193) [23,25,73-75]. Investigations addressing the chemistry behind this phenomenon have led to cumulative evidence indicating the formation of a permanent covalent bond between human as well as selected bacterial DNMTs and 5-azacytidine that can trap the enzyme in a suicide complex (triggering apoptosis). In comparison, only a stable but no permanent covalent bond has been proven between zebularine and the same DNMTs, which would allow the enzymes’ release after binding *in vitro* as well as *in vivo*. This may explain why higher concentrations of zebularine are necessary for similar levels of global DNA hypomethylation in cell nuclei and its lower cytotoxicity (at equimolar concentrations), compared with AZA [76,77].

Furthermore, we observed that the increase in cytotoxicity correlates with global 5-methylcytosine levels, especially the extent of DNA hypomethylation at DAPI-positive heterochromatic sites as revealed by 3D-qDMI through scatter plotting of MeC/DAPI codistribution. This was also true for AZA-treated cells (data not shown). Along the same lines, when localizing low-intensity MeC and DAPI sites in the same nuclei, we could map the gradual increase in LIMs from the nuclear periphery into the more interior parts of the nuclei. However, we experienced that a strong level of LID increase within the nuclei interior was already seen at the lower zebularine concentration (8–40 μM), compared to naïve cells, which did not significantly change up to the highest concentration applied (1000 μM). These LID-patterns were very similar to the one in AZA-treated cells (Figure 8), in which the majority of LIDs were found to be located in the nuclear periphery. These conclusions are drawn from images of cells with seemingly intact nuclear envelope. In fact, for drug concentrations ≥5 μM for 5-azacytidine and ≥500 μM for zebularine, a large number of cells were found to present DAPI and MeC signals outside of their nuclei, leading to the assumption that the drugs had also affected the nuclear envelope and caused DNA leakage. In these cells the respective LID curves were located below the diagonal of the graphs (not shown). Due to the cytotoxic effect induced by high drug concentrations, such cells were not included in our further analyses.

Therefore we cannot exclude any contribution of topological changes of gDNA/heterochromatin to cytotoxicity. On the contrary, we assume that global DNA demethylation may lead to both DNA hypomethylation as well as gDNA reorganization, which are bilateral and together could lead to cellular decline. Although, our data here suggest that cytotoxicity is more fine-correlated with DNA hypomethylation than with bulk DNA reorganization. However, it may also be possible that only local gDNA rearrangements occurred under the conditions applied in our study. The latter effect is conceivable from the increase of LIMs in nuclear areas that harbor heterochromatin: as a significant LIM increase was detected for cells already at low zebularine doses, a compounding of both DNA demethylation effects may have triggered cellular disruption. Figure 6 underlines the fact that 5-azacytidine has equivalent effects at concentrations that are much lower than of zebularine.

The mode of action of azanucleosides is quite complex [78]. Cytosine hypomethylation by azanucleosides, including zebularine, has been extensively reported to reactivate tumor suppressor genes and apoptosis-related genes [79-81] but also the relaxation of highly compacted chromatin that can be seen as a loss of gDNA (DAPI) signal per voxel [43,44], as chromatin conformation is linked to DNA methylation and its bilateral relationship to histone tail modifications [82]. Therefore, we believe that cell-by-cell topological analysis as used in our approach, i.e. the topology of LIMs and LIDs in combination with the display of differential MeC/DAPI colocalization patterns shows a potential to serve as a valuable indicator for the observed phenomena: cytotoxicity-correlated global DNA hypomethylation and DNA reorganization, as consequences of drug effects. For the selected combinations of cell types and agents, the measurement of mean MeC signal (*I*_MeC_) — a derivative of DNA methylation load, across all imaged cells — corresponded well with the level of cytotoxicity (Figure 4). However, for the majority of cases *I*_MeC_ presented a relatively high standard deviation, whereas for the same cell populations we observed a low fraction of dissimilar cells in terms of MeC/gDNA distribution (Figure 5). The discrepancy between the two signatures becomes more plausible with the analysis of synchronized DU145 cells: high similarity was measured between G0/G1-cells and G2-cells in MeC/DAPI codistribution (Figure 10). On the contrary, individual intensity values for global 5-methylcytosine (MeC) and overall DNA (DAPI) nearly doubled between G0/G1-phase and G2-phase as expected, although with a large spread in both signal distributions indicating high signal variability even in synchronized cells (Figure 11). Based on these findings, we believe that signatures based on spatial MeC/DAPI codistribution are more robust in MeC-phenotyping of cells than simply measuring DNA methylation loads, as they can better distinguish between drug-induced demethylation effects and the variation of methylation among individual cells. In combination with K-L divergence measurement, such a cell-by-cell cross-examination as performed with 3D-qDMI can provide structure-based quantities for studying epigenetic drug response.

Finally, in order to test the quantitative accuracy of 3D-qDMI a comparative analysis was performed utilizing MethyLight assays that have been specifically designed for and proven to measure differential levels of DNA methylation in repeat sequences such as Alu, Sat2, and Satα with high confidence [65]. These sequences are highly methylated in human cells and also represent a significant portion of their genomes. Therefore, they have been proven to serve as surrogates for measuring the global content of 5-methylcytosine in cells. Our comparative analyses revealed a significantly high degree of correlation between the outcomes of the two methods. We chose MethyLight as a validated technique over high-pressure liquid chromatography (HPLC), used as a standard method for measuring global DNA methylation: as the latter method requires significantly more input DNA (5–10 μg).

We conclude that the results of our work strongly support the idea of utilizing the spatial higher-order genome organization as a sentinel for drug-induced toxicity effects in liaison with global DNA hypomethylation. In particular, nuclear DNA methylation distribution patterns have proven to serve as an indicator of topological changes of the genome that could perturb spatial interactions of genomic loci and subsequent expression programs leading to cytotoxicity in treated cells. This is quite conceivable as it has been observed that DNA hypomethylation after treatment with DNMTi can be accompanied by additional changes in histone-tail modifications and nucleosome depletion that decrease DNA-repressive mechanisms and support a more open chromatin conformation [83-86], an effect that we could reconcile with 3D image analysis for H3K9me3. A decrease in this repressive and compacting chromatin landmark with increasing doses of 5-azacytidine correlates well with a decrease in gDNA signal, and could be interpreted as chromatin decondensation (Figure 7). These downstream effects remain to be evaluated by determining the underlying molecular effects of possible cellular reprogramming, including the degree of heterochromatin demethylation [87]. Especially, the loss of global DNA methylation at heterochromatic areas of the genome that harbor highly repetitive DNA sequences such as highly abundant Alu repeats, transposable long interspersed nuclear elements (LINEs) and satellite DNAs can be associated with multiple risks towards genome instability [8,88]; through an adverse reorganization of the genome with side effects, such as transcriptional activation of oncogenes, activation of latent retrotransposons, chromosomal instability, and telomere elongation of chromosomes [11,89-92]. More specifically, Satα and Sat2 DNA hypomethylation may favor centromeric and pericentromeric instability, respectively. Alu retroelements, if left unchecked, would insert throughout the genome into non-coding and coding regions. The result would be mutations, and activation of oncogenes: spontaneous insertion of an Alu element causes nearby promoters to be hypomethylated, increasing gene expression [93,94]. Diseases directly associated with Alu insertion into coding regions include neurofibromatosis, haemophilia, agammaglobulinaemia, leukemia, breast cancer and ovarian cancer [95]. Any malignancy caused by Alu insertion is both heritable along somatic cell lines as well as in the germline. This concern has been recently strengthened by observations, in which specific genomic areas were found to become re-methylated during a following DNA replication step after initial drug-induced demethylation; as a possible mechanism to protect these sequences from permanent hypomethylation [96]. The study showed that exposure of cancer cells to agents such as 5-azacytidine and decitabine preferentially led to demethylation of CpGs not located in CpG-islands, whereas island-associated CpGs became preferentially re-methylated, suggesting that CG-dinucleotides in repetitive elements could become more persistently hypomethylated than gene-associated CGs.

## Conclusions

In light of these observations, it appears reasonable to point out the necessity of new assays and complementary bioinformatics for detecting unwanted genomic-scale adverse effects such as heterochromatin reorganization, that could be used as endpoints in the cytotoxic and genotoxic risk assessment of already existing demethylating drugs and next-generation chromatin-targeting agents under development. Recent advancements in cellular imaging and computational image analysis have made it feasible for large volumes of images from thousands of cells to be analyzed in relatively short amount of time at substantially lower costs. Imaging-based cytomics also enables the quantification of spatial and temporal distribution of molecules and cellular components within their native environment [97], which can boost understanding drug activity at the cell systemic level. Within this context, MeC phenotyping appears to provide a valuable technology, and further investigations will be crucial to evaluate its performance for a broader spectrum of epigenetic drugs in cytotoxicity and eventually genotoxicity testing. Hence, the combination of 3D-qDMI with comparative techniques that provide genome-wide sequence-specific MeC-profiles and detail concurrent changes in chromatin conformation could lead to validation of MeC phenotypes in assessment of drug-induced chromatin states. A variety of impressive high-resolution sequencing-based techniques have recently become available such as NOMe-Seq [39], which provides nucleosome positioning landscapes; chromosome conformation capture (3C) methodology and its whole-genomic version Hi-C, which map the 3D architecture of the genome by proximity-based ligation and subsequent next-generation sequencing [98,99]; a related method called chromatin interaction analysis using paired-end tag sequencing (ChIA–PET) [100], and newer attempts that focus on increasing the sensitivity of chromatin immunoprecipitation-based assays towards single-cell analysis [101]. For example: the correlation of chromatin textures derived from MeC patterns with matching nucleosome depleted regions and proximity-ligation profiles can lead to the identification of MeC phenotypes indicative of risky and genotoxic drug effects.

## Abbreviations

CSMC: Cedars-Sinai Medical Center; Cy3: Cyanine 3; FBS: Fetal bovine serum; IC_10_: Inhibitory concentration at which 10% of cells are nonviable.

## Competing interest

The authors declare that they have no competing interests.

## Authors’ contributions

JT designed and conducted the study, and performed all drug experiments. JHO contributed with cell synchronization assays. KAW performed imaging. AG and JT conceptualized image analyses. AG contributed analytical software tools. AG and JT performed image and statistical data analysis. JT wrote the manuscript together with AG. DJW was in charge of the comparative MethyLight assays and helped with manuscript revision. All authors read and approved the final manuscript.

## Pre-publication history

The pre-publication history for this paper can be accessed here:

http://www.biomedcentral.com/2050-6511/14/11/prepub
